# Sexually Transmitted Infections Prevalence and Cascade of Care among Undocumented Sex Workers: A Twenty-Year-Long Experience

**DOI:** 10.3390/life13030606

**Published:** 2023-02-22

**Authors:** Alessandra Donisi, Agnese Colpani, Beatrice Zauli, Andrea De Vito, Vito Fiore, Sergio Babudieri, Giordano Madeddu

**Affiliations:** 1 Migration Health Unit, Primary Health Care Department, AUSL Piacenza, 29121 Piacenza, Italy; 2 Unit of Infectious Diseases, Department of Medicine, Surgery and Pharmacy, University of Sassari, 07100 Sassari, Italy

**Keywords:** sexually transmitted infections, sex workers, undocumented migrants, STI, screening

## Abstract

Undocumented migrant sex-workers (SW) are vulnerable to Sexually Transmitted Infections (STIs). However, data regarding prevalence and linkage-to-care are lacking. Defining epidemiology is crucial to implement preventive measures. We report data from SW attending a facility for migrants in Piacenza, Italy. We collected medical records from 1999 until 2021. Quantitative variables were summarized as mean and standard deviation (SD), and qualitative ones by absolute and relative frequencies. Logistic regression analysis was performed to assess the relationship between sociodemographic, clinical variables, positive testing, and loss to follow-up (LFU). Overall, 1035 STI episodes were collected, 917 in cisgender-females (CF), and 118 in transgender-females (TF). Overall, 474 diagnoses were made. Three-hundred-ninety-two/474 (82.7%) started therapy, and 264/474 (55.7%) complied with a follow-up. Only 51.5% of HBV and 30.8% of HIV were linked to care. Having symptoms (OR 1.70 (95% CI 1.06–2.73), *p* = 0.028) and previous STIs (OR 1.36 (95% CI 1.04–1.77), *p* = 0.022) were associated with a higher chance of STIs, while at-risk intercourse to lower risk (OR 0.19 (95% CI 0.07–0.49), *p* = 0.001). TF had higher odds of bloodborne infections and syphilis (OR 2.61 (95% CI 1.17–5.80), *p* = 0.019). Regarding follow-up, the older the patient, the higher the LFU (OR 1.05 (95% CI 1.01–1.10), *p* = 0.021). Our data showed a high prevalence of STIs and LFU among undocumented SW. TF are even more vulnerable. Further efforts should be put into targeted interventions.

## 1. Introduction

According to the World Health Organization (WHO), more than one million Sexually Transmitted Infections (STIs) are acquired every day around the world [1]. In Italy, STIs have been increasingly reported; data from the Italian National Institute of Health confirm that from 2005 to 2019, STI notifications have increased by 41.8% compared to the period 1991–2004, with a four-fold raise in cases between 2008 and 2019 [2].

Men who have sex with men (MSM), adolescents/young adults, sex workers (SWs), and their clients are at risk populations for acquiring STIs [3,4].

Considering precarious living conditions, discrimination, and poor access to care, undocumented migrants who work as SWs are particularly vulnerable to STIs, late diagnosis, and deceptive linkage to care [5,6,7]. Additionally, a focus on migrants and undocumented migrants is advocated by the European Centre for Disease Prevention and Control (ECDC) [8]. However, health equity in Europe is far from being reached. In a survey conducted among 1407 migrants from ten European countries, Italy was among the worst countries for perceived discrimination in a medical setting; also, female migrants had higher odds of needing, and not having access to, health services than their male counterparts [9].

Female migrant SWs are a particularly at-risk population and are too often neglected. Data on prevalence and linkage to care of this population are lacking. A recent review by McBride et al. reported interesting data regarding migrant SWs worldwide. Among 29 studies included, six were from Europe and one from Italy. A link between precarious immigration status and poorer HIV and STI outcomes was highlighted. Many studies in the field provide information regarding STI prevalence among migrant SWs. However, most studies focus on bloodborne infections and syphilis and do not report other STIs [10,11,12].

Therefore, a mobile population’s long-term follow-up and continuum of care are critical issues. Regarding Italy, a study conducted in 2014 to assess the continuum of care for people diagnosed with HIV highlighted that being a migrant was associated with higher odds of not being on stable antiretroviral therapy and not being virologically suppressed [13]. One of the barriers to the continuum of care is the lack of a personal Electronic Health Record for migrants living in Italy; thus, when they move to different cities, previous medical history is often lost. Nevertheless, few studies conducted among migrant sex workers report a cascade of care and long-term follow-up.

For this reason, we decided to conduct a retrospective study for more than 20 years among undocumented migrant SWs in Piacenza, Italy. We aimed to describe the epidemiology of STIs and the cascade of care among this population and evaluate possible risk factors for acquiring STIs and for not adhering to treatment and follow-up. Our work could help identify critical issues that may be targeted by interventional campaigns to improve testing, treatment, follow-up, and prevention in a difficult-to-reach population.

## 2. Materials and Methods

### 2.1. Data Collection

We collected medical records from the Migration Health Unit, Piacenza, Italy. Data from 1999 until 2021 were collected. Only self-defined SWs were included. They have been referred to the outpatient clinic thanks to an “on-the-road” project conducted by Piacenza social services. In Piacenza, SWs are recipients of a project called “Oltre la Strada” (Beyond the Street). Social services constantly work on the road to offer SWs legal, psychological, and medical assistance. Undocumented migrant SWs are then addressed to the local outpatient clinic for migrants. Each clinical visit is conducted with the support of proper cultural mediation. Demographics, anamnesis, previous abortions, voluntary termination of pregnancy (VTP), drug or alcohol habits, reasons for examination, diagnostic tests, results, treatment, and follow-up schedule were collected from the clinical chart. Follow-up visits were recorded as part of the same episode. However, if a patient missed treatment or follow-up and was accessed for a new visit, a new episode was recorded. Two young expert physicians (AC and BZ) examined paper medical records under the supervision of a senior expert physician (AD).

The research was conducted according to the Helsinki Declaration. The research does not contain clinical studies, and all participants’ data are fully anonymized. For this type of study, formal consent is not required according to current national law from Italian Medicines Agency, and according to the Italian Data Protection Authority, neither Ethical Committee approval nor informed consent was required for our study.

We use the term cisgender females (CF) to refer to women who were assigned female at birth and transgender females (TF) to women who were assigned male at birth and underwent a transition process.

The screening included HIV, HBV, HCV, and syphilis for all patients; *Chlamydia trachomatis*, *Neisseria gonorrhea*, *Ureaplasma urealyticum/parvum,* and *Mycoplasma genitalyum/hominis* testing were offered only to cisgender-females (CF).

### 2.2. Laboratory Analysis

- HIV, HBV, and HCV were tested with chemiluminescent immunoassay (CLIA) or enzyme-linked fluorescence assay (ELFA) methods;
- Syphilis was tested with the CLIA method, and if positive, it was confirmed by *Treponema pallidum* Hemagglutination Assay (TPHA) and rapid plasma regain (RPR);
- Before 2015 *Chlamydia trachomatis*, *Neisseria gonorrhea*, *Ureaplasma urealyticum/parvum*, and *Mycoplasma genitalyum/hominis* were tested on cervical swab by cell culture;
- After 1 January 2015, Multiplex Polymerase Chain Reaction (PCR) on cervical swab was used to diagnose *Chlamydia trachomatis*, *Neisseria gonorrhea*, *Ureaplasma urealyticum/parvum* and *mycoplasma genitalyum/hominis*.

When a microorganism was identified, antimicrobial susceptibility testing was provided.

Proper treatment was decided by the infectious diseases specialist who is in charge of the outpatient clinic. In addition, counselling regarding prevention and routine testing was offered to all patients.

### 2.3. Data Analysis

Quantitative variables were summarized as mean and standard deviation (SD); qualitative ones by absolute and relative frequencies. Differences were evaluated by Student *t*-test, Pearson Chi-Square, or Fischer exact tests as appropriate. Logistic regression analysis was performed to assess the relationship between sociodemographic, clinical variables and diagnosis, adherence to treatment, and loss to follow-up (LFU). A two-tailed *p*-value less than 0.05 was considered statistically significant. All statistical analyses were performed with STATA version 16.1 (StatsCorp, Prosper, TX, USA).

## 3. Results

After examining all the records of SWs, no refusal to undergo the screening was reported. However, some reluctance to undergo urethral swabs was shown by TF, who were only routinely offered serological screening unless they referred symptoms that required swab testing. Overall, 1035 episodes were collected, of which 917 occurred in CF and 118 in TF. Characteristics of participants are reported in Table 1.

**Figure 1:**
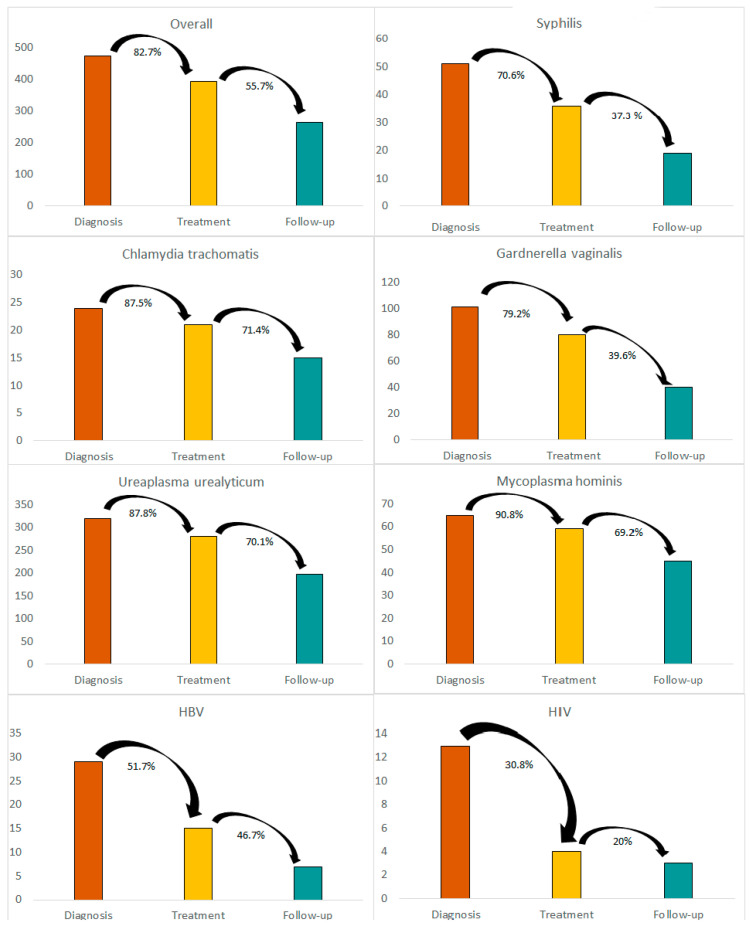
Cascade of care for each Sexually Transmitted Infection diagnosed among undocumented migrant sex workers between 1999 and 2021 in Piacenza, Italy.

Seven-hundred-nineteen cervical swabs, 958 syphilis, 953 HIV, 936 HCV, and 938 HBV screenings were performed. Overall, 474 (45.8%) episodes resulted in the isolation of at least one pathogen. Three-hundred-ninety-two/474 (82.7%) patients came back to start therapy, and 264/474 (55.7%) complied with follow-up (Figure 1). Overall, the most common diagnosis was *U. urealyticum* (n = 320, 30.9%), followed by *G. vaginalis* (n = 101, 9.8%), *M. hominis* (n = 65, 6.3%) and syphilis (n = 51, 4.9%). Regarding viruses, HBV was the most common (n = 29, 2.8%), followed by HIV (n = 13, 1.3%). Of interest, only 51.5% of HBV and nearly 30.8% of HIV diagnoses were linked to care. The cascade of care for each diagnosis is shown in Figure 1.

Risk factors associated with a diagnosis of STI are reported in Table 2.

Having symptoms (OR 1.70 (95% CI 1.06–2.73), *p* = 0.028) and reporting previous STIs (OR 1.36 (95% CI 1.04–1.77), *p* = 0.022) were associated with a higher chance of receiving a diagnosis, while at risk intercourse as a reason for testing showed the opposite (OR 0.19 (95% CI 0.07–0.49), *p* = 0.001). Being a TF was associated with a lower risk of receiving a diagnosis (OR 0.56 (95% CI 0.35–0.90, *p* = 0.016). However, since they were tested only for syphilis and blood born infection, we performed a sub-analysis considering only these diagnoses (See Supplementary Materials, Table S1). The results show that being a TF was associated with higher odds of receiving a diagnosis (OR 2.61 (95% CI 1.17–5.80*), p* = 0.019), as well as ongoing pregnancy (OR 10.41 (95% CI 1.76–61.6), *p* = 0.010) and having symptoms (OR 4.38 (95% CI 0.83–23.07), *p* = 0.081).

Regarding predictors of treatment adherence, having a diagnosis of HIV (OR 12.77 (95% CI 1.17–161), *p* = 0.037), and HBV (OR 4.66 (95% CI 1.28–16.9)*, p* = 0.019) were associated with non-adherence. In contrast, being diagnosed with *U. urealyticum* [(OR 0.26 (95% CI 0.15–0.45)*, p* < 0.001)], was associated with increased compliance to treatment (Table 3).

Finally, regarding follow-up, the older the patients, the higher the risk of LFU (OR 1.05 (95% CI 1.01–1.10), *p* = 0.021). In addition, being Brazilian (OR 4.60 (95% CI 1.02–20.82)*, p* = 0.048) and HBV diagnosis (OR 4.04 (95% CI 1.13–14.5), *p* = 0.032) were associated with poorer compliance (See Supplementary Materials, Table S2).

## 4. Discussion

STIs represent a serious health problem around the world. The Global Health Sector Strategy on Sexual Transmitted Infections of the WHO encourages early diagnosis by screening asymptomatic at-risk individuals [1]. In particular, the WHO outlines SWs, migrants, and mobile populations as those usually left behind by surveillance programs.

Several studies have been performed to assess the prevalence of STIs among this neglected population.

A recent systematic review by McBride et al. includes 29 studies, six from Europe and one from Italy. The authors link precarious immigration status with poorer HIV and STI outcomes. Moreover, they point out stigma and criminalization as barriers to sexual health access. In this review, HIV prevalence ranges from 0.3 to 13.6% among immigrant SWs, while we report a prevalence of 1.3%. Of notice, our study includes only migrants in criminalized sex work, and most of them were undocumented at the time of testing, which are two conditions leading to precarious working conditions, and delayed access to care [10]. Mc Bride et al. report syphilis as the primary STI diagnosis, while we report *U. urealyticum*, followed by *G. vaginalis*, and *M. hominis* as the most common. Nevertheless, a screening of these pathogens was performed in only a few studies.

Regarding data from Italy, a study conducted in Verona from 1999 to 2007 among migrant SWs reports an HIV prevalence of 4.6% and a syphilis prevalence of 2.0% (compared to 1.3% and 4.9%, respectively in our study) [12]. Considering the different time-span of the two studies, these data could reflect European and national trends for syphilis and HIV [14,15], which show a rising trend for the first and a decrease for the latter [15].

Regarding other European studies, in 2011, Dias et al. conducted an HIV screening among female SWs in Portugal, reporting 0.8% among undocumented and 1.8% among documented migrants, which is similar to our findings, taking into account that we also included TFs [11]. Verhaegh-Haasnoot et al. conducted a screening of syphilis, chlamydia, gonorrhoea, HIV, and HBV in the Netherlands from 2007 to 2012. They report a prevalence of 40% of STIs among male SWs, 9% among female SWs, and 14% among men who have sex with men (MSM) SWs. However, these data are difficult to compare to ours since sex industry policies are entirely different in Italy and the Netherlands. Moreover, they targeted SWs, regardless of their migration status and citizenship [16].

Regarding HBV, we found a higher prevalence than estimated among the general Italian population (2.8% vs. 0.7%) [17] but lower than the prevalence reported in Verona among female migrant SWs (2.8% vs. 3.5%) [12]. These data reinforce the European call for vaccination and prevention among an under-immunized population, as migrants often are [8].

Few controversial data regarding *U. urealyticum* and *M. hominis* are available in the literature [18]. Some data suggest that these pathogens may contribute to male and female infertility [19], and adverse pregnancy outcomes [20]. However, a position statement by Horner et al. discourages routine testing for *U. urealyticum* and *M. hominis* in asymptomatic individuals [21]. Furthermore, the authors state that indiscriminate testing can be a burden more than good in certain settings. At the same time, they do not consider specific at-risk populations, such as the one examined in our study. In our study, we report 320 (30.9%) diagnoses of *U. urealyticum* and 65 (6.3%) diagnoses of *M. hominis*. A study conducted among Italian females from the general population reports a prevalence of 9.0% for *U. urealyticum* and 8.6% for *M. hominis*. The higher prevalence of *U. urealyticum* among a population of SWs supports its transmission through sexual intercourse, as previously suggested [22]. Further data may be useful to establish the cost-effectiveness of testing and treatment of this pathogen among selected populations.

Limited data are reported in the literature concerning TFs [10]. Our study supports their susceptibility to bloodborne infections and syphilis. Behavioral changes through educational programs are necessary to reduce the prevalence of STIs among them. Patients should be educated about the use of barrier methods, which should be easily available for everyone. Moreover, being Brazilian was linked to a higher loss of follow-up. This variable was co-linear with being a TF since 97.5% of TFs included in our study were Brazilian. Thus, not only TFs are more vulnerable to STIs, but they must be considered at higher risk of LFU. This could be due to self-stigma, anticipated stigma, and perceived discrimination, especially when HIV is diagnosed [23]. In this regard, training of medical staff is fundamental to improving retention in care [24].

Regarding linkage to care, we highlight a dramatic LFU of patients diagnosed with HIV, with only 30.8% of patients starting treatment and 20.0% adhering to follow-up. Data from Rwanda [25] and Uganda [26] report higher compliance to treatment and follow-up (87% and 79%, respectively); however, these data refer to a sedentary population of SWs. A study conducted in Canada confirms a scarce linkage to care in asylum seekers diagnosed with HIV, with only 43% of new diagnoses linked to care within 30 days [27]. However, we examined data from undocumented migrants, whose living conditions are often more precarious and ever-changing.

Regarding HBV, only 51.7% of patients underwent confirmation analysis and started treatment, and 41.7% attended follow-ups. A study in Belgium suggests that point-of-care testing could be a successful strategy to improve compliance with follow-up [28]. As previously discussed, we think that linkage to care for chronic diseases is incredibly challenging among a mobile population.

Furthermore, concerning is the data regarding syphilis, with 70.6% of patients adhering to treatment, only 37.3% showing up for follow-up visits. Higher compliance to treatment can be easily explained since the first line of treatment is injective penicillin, administered the same day of the diagnosis or presumptive diagnosis, however, sometimes a three-dose course was required, which could explain the LFU.

Data regarding bacterial STIs, *C. trachomatis*, *U. urealyticum* and *M. hominis* diagnoses are more reassuring since they showed a high adherence to treatment (87.5–90.8%) and attendance to follow-up (around 70%). Regarding *G. vaginalis*, compliance is lower, with only 39.6% of patients presenting for follow-up visits. Antibiotic treatment was given the same day of the diagnosis, this could explain the optimal compliance. In addition, acute STIs are more likely to provoke symptoms, this could lead to a will of recovery. Moreover, curable bacterial STIs are easier to accept and manage than HIV, HCV, and HBV.

Our study has some limitations. Since it is a retrospective study and a single-center experience, it may not reflect the European or Italian situation. However, we think that any occasion in a difficult-to-reach population is precious to be shared going in the direction of improving health access all over the world, as advocated by the United Nations [29]. Points of strength include the wide time frame covered and the large sample size. Furthermore, we included TFs, who are frequently left behind. Our data show a substantial risk of contracting STIs among this population, which should be carefully considered for targeted campaigns. Finally, we found particularly interesting the data regarding LFU, which is a critical issue to consider, and scarcely discussed in the literature. Data regarding the cascade of care highlight the need for implementing health access and a continuum of care for a highly mobile population.

## 5. Conclusions

In conclusion, our data show a high prevalence of STIs among undocumented migrant SWs, confirming the need for free and inclusive health facilities for marginalized populations. Moreover, all SWs attending the outpatient clinic accepted to undergo STI screening, encouraging us to keep offering this kind of service. Regarding viral STIs, our data show a dramatic LFU. Improving linkage to care must be crucial in clinicians’ agenda, especially among vulnerable populations. This could be achieved through proper facilities, including cultural mediation, a non-judgmental approach, and point-of-care testing (primarily addressed to mobile populations). Among at-risk populations TFs, represent an even more vulnerable group. More efforts should be made to design targeted interventions addressing this population.

A prompt diagnosis, early treatment, and retention in care for chronic diagnoses improve individual health and prevent disease spreading with a benefit for the whole community. 

## Figures and Tables

**Table 1:** Characteristics of 1035 sex workers attending the outpatient clinic for migrants in Piacenza from 1999 to 2021, according to gender.

	Overall (1035)	Cisgender-Females (917, 88.6%)	Transgender Females (118, 11.4%)	*p*-Value
Age, mean (±SD)	26.2 ± 5.9	26.2 ± 5.6	29.5 ± 7.4	<0.001
Nation of Origin, N (%)	N (%)	N (%)	N (%)	
Nigeria	659 (63.7)	658 (71.8)	1 (0.8)	<0.001
Brazil	123 (11.9)	8 (0.9)	115 (97.5)	<0.001
Albania	32 (3.1)	32 (3.5)	0 (0.0)	0.039
Romania	88 (8.5)	88 (9.6)	0 (0.0)	<0.001
Ecuador	36 (3.5)	35 (3.8)	1 (0.8)	0.098
Rest of Center and South America	18 (1.7)	17 (1.9)	1 (0.8)	0.431
Rest of Africa	25 (2.4)	25 (2.7)	0 (0.0)	0.069
Resto of Europe	52 (5.0)	52 (5.7)	0 (0.0)	0.008
Asia	2 (0.2)	2 (0.2)	0 (0.0)	0.612
Medical history, N (%)	N (%)	N (%)	N (%)	
Previous induced abortion	623 (60.2)	623 (67.9)	0 (0.0)	<0.001
Previous STIs	403 (38.9)	342 (37.3)	61 (51.7)	0.002
Habits, N (%)	N (%)	N (%)	N (%)	
Smoker	306 (29.6)	215 (23.4)	91 (77.1)	<0.001
Alcohol	444 (42.9)	385 (42.0)	59 (50.0)	0.098
Recreative drugs	26 (2.5)	3 (0.3)	23 (19.5)	<0.001
PWID	42 (4.1)	5 (0.5)	37 (31.4)	<0.001
Reason for testing, N (%)	N (%)	N (%)	N (%)	
Screening	871 (84.2)	774 (84.4)	97 (82.2)	0.537
Ongoing pregnancy	45 (4.3)	45 (4.9)	0 (0.0)	0.014
At risk intercourse	37 (3.6)	27 (2.9)	10 (8.5)	0.002
Symptoms	80 (7.7)	70 (7.6)	10 (8.5)	0.747
Type of test, N (%)	N (%)	N (%)	N (%)	
Swab Negative Positive Not performed	321 (31.0) 398 (38.5) 316 (30.1)	317 (34.6) 397 (43.3) 203 22.1)	4 (3.4) 1 (0.8) 113 (95.8)	
Syphilis screening Negative Positive Not performed	856 (82.7) 102 (9.9) 77 (7.4)	803 (87.6) 44 (4.8) 70 (7.6)	53 (44.9) 58 (49.2) 7 (5.9)	<0.001
HIV screening Negative Positive Not performed	937 (90.1) 16 (1.5) 81 (7.8)	840 (91.6) 4 (0.4) 73 (8.0)	98 (83.1) 12 (10.2) 8 (6.8)	<0.001
HCV screening Negative Positive Not performed	930 (89.9) 6 (0.6) 94 (9.1)	831 (90.6) 6 (0.7) 80 (8.7)	104 (88.1) 0 (0.0) 14 (11.9)	
HBV screening Negative Positive Not performed	906 (87.5) 32 (3.1) 93 (8.9)	806 (87.9) 32 (3.5) 79 (8.6)	103 (87.3) 1 (0.8) 14 (11.9)	0.172
Diagnosis, N (%)	N (%)	N (%)	N (%)	
Chlamydia	24 (2.3)	24 (2.6)	0 (0.0)	0.075
Gardnerella vaginalis	101 (9.8)	101 (11.0)	0 (0.0)	<0.001
Gonorrhoea	4 (3.9)	3 (0.3)	1 (0.8)	0.391
Syphilis	51 (4.9)	22 (2.4)	29 (24.6)	<0.001
Ureaplasma	320 (30.9)	320 (34.9)	0 (0.0)	<0.001
Mycoplasma	65 (6.3)	65 (7.1)	0 (0.0)	0.003
Trichomonas	8 (0.8)	8 (0.9)	0 (0.0)	0.308
HBV	29 (2.8)	27 (2.9)	2 (1.7)	0.439
HCV	6 (0.6)	6 (0.7)	0 (0.0)	0.378
HIV	13 (1.3)	3 (0.3)	10 (8.5)	<0.001
HPV	5 (0.5)	4 (0.4)	1 (0.8)	0.544

SD: standard deviation; STIs: sexually transmitted infections; PWID: people who inject drugs.

**Table 2:** Variables associated with risk of receiving a diagnosis of STI among 1035 undocumented migrant sex workers in Piacenza, Italy.

	OR (95% CI)	*p*-Value	aOR (95% CI)	*p*-Value
Age, mean (SD)	0.99 (0.96–1.01)	0.229		
Transgender female	0.48 (0.31–0.72)	<0.001	0.56 (0.35–0.90	0.016
Nigerian	0.85 (0.66–1.1)	0.204		
Brazilian	0.47 (0.31–0.70)	<0.001		
Albania	1.35 (0.67–2.74)	0.400		
Romania	1.80 (1.15–2.80)	0.01	1.83 (1.16–2.91)	0.010
Ecuador	2.44 (1.21–4.93)	0.013	2.27 (1.19–4.70)	0.027
Rest of South America	0.75 (0.29–1.94)	0.554		
Rest of Africa	1.80 (0.80–4.05)	0.155		
Rest of Europe	1.52 (0.87–2.67)	0.141		
Asia	1.18 (0.07–18.98)	0.905		
Alcohol	0.86 (0.67–1.10)	0.239		
Use of recreative drugs	1.01 (0.46–2.21)	0.971		
PWID	0.64 (0.33–1.21)	0.174		
Primary level of education	1.14 (0.89–1.47)	0.276		
Previous induced abortion	1.31 (1.021–1.68)	0.034	1.15 (0.88–1.54)	0.353
Previous diagnosis of STIs	1.37 (1.07–1.77)	0.013	1.36 (1.04–1.77)	0.022
Reason for testing				
Screening	1.20 (0.85–1.68)	0.297		
Ongoing pregnancy	0.58 (0.31–1.09)	0.090	0.60 (0.32–1.14)	0.120
At risk intercourse	0.18 (0.07–0.46)	<0.001	0.19 (0.07–0.49)	0.001
Symptoms	1.76 (1.11–2.79)	0.017	1.70 (1.06–2.73)	0.028

STIs: sexually transmitted infections; SD: standard deviation; PWID: people who inject drugs.

**Table 3:** Variables associated with loss to follow-up before treatment among undocumented sex workers diagnosed with at least one STI.

	OR (95% CI)	*p*-Value	aOR (95% CI)	*p*-Value
Age, mean (SD)	1.01 (0.98–1.05)	0.527		
Transgender females	3.35 (1.65–6.79)	<0.001	1.78 (0.57–5.59)	0.321
Nigerian	0.73 (0.46–1.15)	0.176		
Brazilian	2.80 (1.38–5.69)	0.004		
Albania	0.89 (0.25–3.15)	0.852		
Romania	0.51 (0.21–1.23)	0.135		
Ecuador	1.77 (0.71–4.40)	0.220		
Rest of South America	0.69 (0.08–5.78)	0.731		
Rest of Africa	1.04 (0.29–3.76)	0.953		
Rest of Europe	1.35 (0.58–3.25)	0.508		
Smoke	1.21 (0.72–2.03)	0.468		
Alcohol	1.08 (0.68–1.71)	0.751		
Use of recreative drugs	2.69 (0.86–8.41)	0.090	1.15 (0.24–5.45)	0.860
PWID	5.97 (2.01–17.65)	0.001	3.98 (0.95–16.6)	0.058
Primary level of education	0.95 (0.60–1.50)	0.818		
Previous induced abortion	0.73 (0.46–1.15)	0.176		
Previous diagnosis of STIs	0.50 (0.30–0.83)	0.007	0.44 (0.25–0.77)	0.004
Reason for testing				
Screening	0.94 (0.49–1.77)	0.841		
Pregnancy	2.14 (0.72–6.14)	0.175		
At risk intercourse	1.04 (0.11–9.4)	0.973		
Symptoms	0.70 (0.30–1.62)	0.412		
Diagnosis				
Chlamydia	0.58 (0.17–1.98)	0.385		
Gardnerella	1.12 (0.65–192)	0.692		
Syphilis	1.87 (0.98–3.59)	0.059	0.22 (0.07–0.68)	0.009
Ureaplasma	0.26 (0.16–0.42)	<0.001	0.26 (0.15–0.45)	<0.001
Mycoplasma	0.38 (0.16–0.91)	0.031	0.42 (0.17–1.03)	0.058
Trichomonas				
HBV	4.77 (2.23–10.15)	<0.001	4.66 (1.28–16.9)	0.019
HCV	21.9 (2.52–189.8)	0.005	1	
HIV	10.2 (3.08–34.07)	<0.001	12.77 (1.17–161)	0.037
HPV	1.04 (0.11–9.40)	0.973		

STIs: sexually transmitted infections; SD: standard deviation; PWID: people who inject drugs.

## Data Availability

The data will be available upon specific request to the Authors.

## Supplementary Materials

The following supporting information can be downloaded at: https://www.mdpi.com/article/10.3390/life13030606/s1, Table S1. Variables associated with a diagnosis of HIV, HCV, HBV, and syphilis, among 1035 undocumented migrant sex workers in Piacenza, Italy. Table S2. Variables associated with loss to follow-up after treatment among undocumented sex workers diagnosed with at least one STI.
