# Supplementary material for: Sexually Transmitted Infections Prevalence and Cascade of Care among Undocumented Sex Workers: A Twenty-Year-Long Experience
Source: Life (Basel). 2023 Feb 22;13(3):606. doi: 10.3390/life13030606 (PMC10056054; doi:10.3390/life13030606)
Supplement: Supplementary file 1 [file life-13-00606-s001.zip › life-2130222-supplementary.pdf]

**Table S1.** Variables associated with a diagnosis of HIV, HCV, HBV, and syphilis, among 1035 undocumented migrants sex workers in Piacenza, Italy.				
	OR (95% CI)	*p*-value	aOR (95% CI)	*p*-value
Age, mean (SD)	1.07 (1.04-1.10)	<0.001	1.03 (0.99-1.07)	0.074
Transgender female	6.34 (3.9-10.28)	<0.001	2.61 (1.17-5.80)	0.019
Nigerian	0.19 (0.12-0.31)	<0.001	0.37 (0.19-0.73)	0.004
Brasilian	5.95 (3.68-9.62)	<0.001		
Albania	1.10 (0.33-3.69)	0.874		
Romania	1.59 (0.81-3.12)	0.176		
Ecuador	1.76 (0.67-4.64)	0.255		
Rest of South America	0.62 (0.08-4.72)	0.645		
Rest of Africa	0.92 (0.21-3.98)	0.914		
Rest of Europe	2.02 (0.92-4.45)	0.079	1.29 (0.52-3.22)	0.582
Asia	1			
Alcohol	1.33 (0.86-2.06)	0.193		
Use of recreative drugs	7.36 (3.23-16.75)	<0.001	2.01 (0.82-5.67)	0.184
PWID	6.86 (3.50-13.46)	<0.001	1.86 (0.77-4.48)	1.70
Primary level of education	2.10 (1.35-3.27)	0.001	1.41 (0.87-2.32)	0.162
Previous induced abortion	0.36 (0.23-0.56)	<0.001	0.97 (0.53-1.79)	0.933
Previous diagnosis of STI	1.31 (0.85-2.03)	0.225		
Reason for testing				
Screening	0.58 (0.34-0.97)	0.038	2.19 (0.46-10.42)	0.322
Ongoing pregnancy	2.43 (1.09-5.38)	0.029	10.41 (1.76-61.6)	0.010
At risk intercourse	1			
Symptoms	1.79 (0.91-3.53)	0.091	4.38 (0.83-23.07)	0.081
SD: standard deviation; PWID: people who inject drugs; STIs: sexually transmitted infections.				

**Table S2.** Variables associated with loss to follow-up after treatment among undocumented sex workers diagnosed with at least one STI.				
	OR (95% CI)	*p*-value	aOR (95% CI)	*p*-value
Age, mean (SD)	1.06 (1.02-1.10)	0.001	1.05 (1.01-1.10)	0.021
Transgender female	2.23 (0.92-5.43)	0.101		
Nigerian	0.50 (0.32-0.76)	0.001	1.19 (0.56-2.55)	0.645
Brazilian	3.22 (1.33-7.81)	0.009	4.60 (1.02-20.82)	0.048
Albania	3.00 (0.98-9.13)	0.053	2.02 (0.53-2.55)	0.645
Romania	1.71 (0.92-3.17)	0.089	1.62 (0.68-3.85)	0.274
Ecuador	0.48 (0.15-1.49)	0.204		
Rest of South America	3.26 (0.59-18.01)	0.176		
Rest of Africa	0.52 (0.14-1.96)	0.337		
Rest of Europe	1.36 (0.57-3.22)	0.490		
Alcohol	1.02 (0.67-1.55)	0.940		
Use of recreative drugs	0.63 (0.12-3.31)	0.590		
PWID	4.10 (0.79-21.4)	0.094	1.09 (0.15-8.15)	0.929
Primary level of education	1.18 (0.78-1.79)	0.429		
Previous induced abortion	0.96 (0.62-1.48)	0.856		
Previous diagnosis of STI	0.70 (0.46-1.07)	0.101		
Reason for testing				
Screening	0.60 (0.34-1.07)	0.083	0.84 (0.42-1.68)	0.632
Ongoing pregnancy	3.87 (0.98-15.19)	0.053	3.59 (0.69-18.67)	0.129
At risk intercourse	0.53 (0.05-5.14)	0.584		
Symptoms	1.35 (0.70-2.61)	0.372		
Diagnosis				
Chlamydia	0.62 (0.23-1.65)	0.341		
Gardnerella	2.07 (1.26-3.41)	0.004	1.74 (0.99-3.07)	0.055
Syphilis	2.16 (1.08-4.31)	0.030	0.36 (0.11-1.24)	0.107
Ureaplasma	0.38 (0.24-0.61)	<0.001	0.64 (0.35 (1.18)	0.152
Mycoplasma	0.60 (0.33-1.10)	0.099	0.68 (0.36-1.32)	0.258
Trichomonas	2.72 (0.64-11.56)	0.175		
HBV	4.67 (1.46-14.96)	0.009	4.04 (1.13-14.5)	0.032
HCV				
HIV	4.87 (0.50-47.31)	0.172		
HPV				
STIs: sexually transmitted infections; SD: standard deviation; PWID: people who inject drugs.				
